# Microbial Production of the Off-Flavor Geosmin in Tilapia Production in Brazilian Water Reservoirs: Importance of Bacteria in the Intestine and Other Fish-Associated Environments

**DOI:** 10.3389/fmicb.2019.02447

**Published:** 2019-10-31

**Authors:** Mie B. Lukassen, Nadieh de Jonge, Sabine M. Bjerregaard, Raju Podduturi, Niels O. G. Jørgensen, Mikael A. Petersen, Gianmarco S. David, Reinaldo J. da Silva, Jeppe L. Nielsen

**Affiliations:** ^1^Department of Chemistry and Bioscience, Aalborg University, Aalborg, Denmark; ^2^Department of Plant and Environmental Sciences, University of Copenhagen, Copenhagen, Denmark; ^3^Department of Food Sciences, Section of Design and Consumer Behaviour, University of Copenhagen, Copenhagen, Denmark; ^4^São Paulo State Agribusiness Technology at Jaú, Jaú, Brazil; ^5^Departamento de Parasitologia, Instituto de Biociências, Universidade Estadual Paulista Júlio de Mesquita Filho, Botucatu, Brazil

**Keywords:** geosmin, *geoA*, off-flavor, Nile tilapia, gastrointestinal tract, aquaculture production

## Abstract

**Aim:**

To determine major sources of microbially produced geosmin in the commercially important aquaculture fish species tilapia.

**Methods and Results:**

Abundance and composition of geosmin-producing bacteria in water and fish biosphere (intestine, digesta, and fins) of Nile tilapia (*Oreachromis niloticus*) raised in net cages in Brazilian freshwater farms were examined. By combining qPCR of the geosmin synthase *geoA* gene and 16S rRNA gene amplicon sequencing to identify potential geosmin-producing organisms, we observed that the proportion and composition of geosmin producers appeared to be rather similar in the water, digesta, intestinal mucous, and on skin, making up about 0.1–0.2% of the total bacterial densities. A high proportion of *Cyanobacteria* and other putative geosmin producers affiliated to the *Actinomycetales* were identified in the intestinal mucous layer. The main uptake site for geosmin in fish is traditionally assumed to be through the gill surface, but the present results suggest that uptake by the intestinal tract may represent a major source of geosmin uptake in fish.

**Conclusion:**

The high abundance of geosmin-producing bacteria in the intestinal mucous layer and digesta may indicate that the digestive system in fish is an important, but hitherto overlooked, source of geosmin and likely other off-flavors in fish.

**Significance and Impact of Study:**

Tainting of fish by microbially produced off-flavors spoils fish quality and lowers consumer preferences for aquaculture-produced freshwater fish. Our results highlight the potential for the application of probiotic microorganisms for management of the intestinal microflora to improve the fish quality.

**HIGHLIGHTS:**

- Off-flavor producing bacteria are widely abundant in aquaculture.
- Off-flavor producers found on skin surface of fish.
- Off-flavor producing bacteria accumulate in the digestive system.
- Off-flavor producers might release significant amounts of off-flavor during lysis in the gut.
- Off-flavor uptake through the digestive system might be quantitatively significant.

## Introduction

Intensive farming of fish in freshwater introduces the risk of spoiling of the fish due to tainting by taste and-odor compounds (TOCs). Several TOCs produced by microorganisms have been identified in fish flesh, e.g., terpenes (Podduturi et al., 2017), but the most commonly known TOC in fish is the earthy smelling terpenoid geosmin (Guttman and van Rijn, 2008; Houle et al., 2011). Due to the low detection threshold of geosmin (<5 ng⋅L^–1^ in water and <250 ng⋅kg^–1^ in fish tissue) by human senses (Omür-Ozbek et al., 2007; Petersen et al., 2011), even small amounts of geosmin in aquaculture-raised fish may cause a negative consumer preference and impact the value of fish products. Tainted fish can be depurated in clean, geosmin-free water, but this procedure requires additional handling and costs (Robertson et al., 2006).

Production of TOCs by microorganisms has been addressed in aquaculture systems using both culture-dependent and culture-independent techniques (Guttman and van Rijn, 2008; Houle et al., 2011; Auffret et al., 2013; Suurnäkki et al., 2015). The geosmin-producing microorganisms have been affiliated to the class *Actinobacteria* and the order *Myxococcales*, as well as to the phylum *Cyanobacteria*, and various fungi (Dickschat et al., 2005; Klausen et al., 2005; Auffret et al., 2011; Bacha et al., 2015). For molecular identification of geosmin-producing organisms, the ubiquitous *geoA* gene, encoding the bi-functional enzyme geosmin synthase (Jiang et al., 2007), has been used as a marker for geosmin-producing microorganisms (Cane and Watt, 2003; Giglio et al., 2008). Quantification of geosmin-producing cells has typically been limited to the water phase, but biofilms and biofilters may also harbor significant numbers of geosmin-producing organisms (Guttman and van Rijn, 2008; Auffret et al., 2011).

In fish, the gills have been assumed to be the major site for uptake of geosmin, as demonstrated by placing the geosmin-producing cyanobacterium *Symploca muscorum* directly on the gill surface of rainbow trout (From and Hørlyck, 1984). After a few minutes, a sensory analysis showed that the flesh had become tainted. Uptake of geosmin through skin, intestine, and stomach has also been proposed as alternative sources, but the time required to detect tainting of the flesh was 15 to 70-fold longer than through the gills (From and Hørlyck, 1984). Thus, uptake and digestion of geosmin-containing cyanobacteria could result in accumulation of geosmin in the fish flesh. Yet, concentrations of dissolved geosmin in water in fish farms are often sufficiently high to cause accumulation of geosmin in the fish flesh above the human threshold level, as observed for both rainbow trout and tilapia (Petersen et al., 2011; Auffret et al., 2013; Gutierrez et al., 2013).

Uptake of dissolved geosmin from the water may not be the only process by which fish become tainted. Microorganisms in the fish gut may also include geosmin producers, as shown by the presence of geosmin-producing *Streptomyces* spp. in the digestive system of tilapia (*Oreachromis niloticus*) in Thai fish farms (Gutierrez et al., 2013). Tilapia is also known to consume and digest cyanobacteria, e.g., attached on cage surfaces (Watson et al., 2016). Many filamentous cyanobacteria are geosmin producers (Watson et al., 2016), and therefore tilapia may ingest geosmin-producing microbes. However, since a portion of the intracellular geosmin in bacteria is bound to membrane proteins (Jüttner and Watson, 2007), it is uncertain to which extent geosmin is released from ingested microbes and may be transported to the flesh. Possibly, enzymatic activity and the low pH in the digestive system (Moriarty, 1973) of the fish may promote release of cell-bound geosmin.

Knowledge of occurrence of geosmin-producing microorganisms in the fish biosphere, e.g., in water surrounding the fish and in the intestine systems, but also fish skin, will provide valuable information on mechanisms controlling off-flavor tainting of fish in aquaculture systems. Here, we aimed to estimate populations of putative geosmin-producing bacteria in the production of Nile tilapia (*Oreachromis niloticus*) in cages in freshwater reservoirs in southeastern Brazil. Abundances of geosmin-producing bacteria were determined by quantitative PCR targeting the *geoA* gene, and total microbial populations were characterized by a 16S rRNA gene amplicon sequencing assay. In an attempt to identify important sites for the geosmin production, microbial communities were examined in the water, in the intestinal mucous layer, in digesta, and on the dorsal fin (representing the fish skin). We hypothesized that ingested bacteria are a potentially important, but hitherto overlooked, source of geosmin in fish from freshwater ecosystems.

## Materials and Methods

This study was carried out in accordance with the principles of the Basel Declaration and recommendations of ethical principles in animal research formulated by the Brazilian Society of Science in Laboratory Animals, Bioscience Institute/UNESP Ethics Committee on use of animals (CEUA; protocol number 724). The protocol was approved by the National Council for the Control of Animal Experimentation (CONCEA).

### Study Sites and Sample Collection

Fish and water samples were collected in six different freshwater reservoirs within rivers dammed and used for hydroelectric power, and also contained an aquaculture fish farm located in the state of São Paulo, Brazil. The sampling sites included the following farms: Chavantes reservoir (farm 1; −23.124 S, −49.626 W), Nova Avanhandava Reservoir (farm 2; −21.190094 S, −50.04982 W; farm 3, −21.060131 S, −50.092291 W; farm 4, −21.110500 S, −50.098792 W), and Ilha Solteira (farm 5, −20.414813 S, −51.255386 W; farm 6, −20.036386 S, −50.931963 W). All farms produced Nile tilapia (*Oreochromis niloticus*) in intensive grow-out systems in floating net cages. The numbers of cages in each farm range from 150 to 400 with a density of 3 to 12 metric tons per cage. The daily feeding rate per farm varies from 3 to 10 tons, and the feed conversion rate is estimated to range from 1.5 to 2.0. In each of the six farms, we randomly selected one cage and caught 10 fish with a handheld net between 9 and 12 AM. The fish had typically been fed about 20 h earlier. The fish were killed by transfer to ice-cold water directly on site. Subsamples (filets) of the fish were cut out in the laboratory before freezing at −20°C. A total of 60 fish were collected.

Water samples were collected in 1 L sterile glass bottles at about 0.5 m depth in 10 cages from each farm. For the microbiological diversity studies, 150 mL of pooled water was filtered on site through 0.45 μm pore size 47 mm diameter polycarbonate filters and stored on ice until freezing at −20°C in the laboratory. For analysis of geosmin, 30 mL water samples were taken in the 10 cages and stored in glass tubes with added NaCl (final concentration of 5% w/vol) and without air headspace. The tubes were kept on ice during transport to the laboratory.

From each of the fish, the dorsal fin (representing the skin), intestinal mucous, and digesta from the small intestine were taken aseptically. Content in the small intestine was obtained with a sterile cell-scraper, while intestinal mucous was removed with a sterile scalpel. The entire intestinal tract was sectioned into three equal fragments for analysis of the longitudinal distribution of potential geosmin-producing microorganisms. The dorsal fin, mucous and content of intestine, and the filtered water samples were stored in RNA*later* RNA Stabilization Reagent (Qiagen) at −20°C until further analyses.

### DNA Extraction

Genomic DNA was extracted from approximately 9 cm^2^ of the dorsal fins and the intestinal mucous using the DNeasy Blood and Tissue kit (Qiagen). DNA in intestinal samples was extracted with the QIAamp Fast DNA stool kit (Qiagen). All extractions were conducted following recommendations by the manufacturer. The polycarbonate filters with particulate material from the water were cut into three equal sections using a sterile scalpel, and DNA was extracted with the FastDNA^TM^ SPIN kit For Soil (MP Biomedicals) as recommended by the manufacturer, except that bead beating was set to 2 × 40 s at 6 m⋅s^–1^. The extracted DNA was quality controlled using the Tapestation 2200 and Genomic DNA ScreenTape (Agilent), while the concentrations were determined with the Quant-iT HS DNA assay (Thermo Fisher Scientific) on an Infinite M1000 PRO plate reader (Tecan, Lifesciences).

### Amplicon Analysis

Bacterial community profiling in samples from cage water, intestine, and dorsal fins was conducted by high throughput sequencing of the V1–V3 region of the 16S rRNA gene. Ten ng of genomic DNA was amplified using the V13 primer set: 27F AGAGTTTGATCCTGGCTCAG and 534R ATTACCGCGGCTGCTGG (Caporaso et al., 2011), in a total reaction volume of 25 μL (2 mU Platinum Taq DNA Polymerase, 1x Platinum High Fidelity buffer (Thermo Fisher Scientific), 400 nM of each dNTP, 1.5 mM MgSO_4_, and 400 nM of each primer fused with Illumina adaptors) in duplicates. Amplicons were validated using Qubit dsDNA High Sensitivity Assay Kit (Thermo Fisher Scientific), and TapeStation 2200 using D1000 ScreenTapes (Agilent), and purified using Ampure XP bead protocol (Beckmann Coulter) using a bead:sample ratio of 0.8. The amplicons were pooled in equimolar concentrations, and the library pool was sequenced on a MiSeq benchtop sequencer (Illumina) using the MiSeq Reagent kit v3 (2 × 300 PE). In total, the microbial composition was determined for water from 76 fish cages, 17 dorsal fins, 13 intestinal content samples, and 87 intestinal mucous samples.

Amplicon bioinformatic processing was conducted as described elsewhere (Ziegler et al., 2016). The results were analyzed in R statistical software (R Core Team, 2016) through the Rstudio IDE^1^. For analysis of relationships between the observed parameters, constrained ordination by redundancy analysis (RDA) and heat maps were conducted using the R packages ampvis2 (Andersen et al., 2018) and ggplot2 (Wickham, 2009). Chao1 indices were used to visualize microbial richness. All sequence data used in this study has been made available at the European Nucleotide Archive (ENA) under the project accession PRJEB33049.

### Quantitative PCR

Numbers of *geoA* copies in water, intestine, and dorsal fins were determined by quantitative PCR (qPCR). Quantitative PCR (qPCR) of the geoA were carried out using the Mx3005P qPCR system (Stratagene) and the EXPRESS qPCR Supermix (Life Technologies). Reactions of 20 μL were prepared according to manufacturer’s instructions using 50 nM ROX, 500 nM of each primer (DNA Technology), 200 nM hydrolysis probe (DNA Technology) and 5 μL template DNA. The qPCR conditions were as follows: UDG incubation (50°C, 2 min) and PCR activation (95°C, 2 min), followed by 45 cycles of denaturation (95°C, 15 s) and combined annealing and extension (60°C, 1 min). The following primers and probes were used: geoA_g1F geoA group 1 AACACCGTGCTCACCGAAAT, geoA_g1R geoA group 1 TCCAAGCCTTCGCATCCA, geoA_g1PR geoA group 1 6-FAM-CCCTTGCTGCAGGACGATCACGA-BHQ-1, geoA_g3F geoA group 3 CGATGCAGGTGCTCAAAGAC, geoA_g3R geoA group 3 GCTGGTAGGAGAACAGGTCGTT, geoA_g3PR geoA group 3 6-FAM-CCTTCTCCGACGGCGT CCACC-BHQ-1, geoA_g4F geoA group 4 GCACACCTGCCGT TCCTAA, geoA_g4R geoA group 4 GAATGGTGCGATTCC ATAGATCTT, geoA_g4PR geoA group 4 6-FAM-ACCCCGTC GAGCGTGCGCT-BHQ-1, geoA_g5F geoA group 5 GCGGCT TCAGCAGTTTGAA, geoA_g5R geoA group 5 GTCCGTACT CCGCACACAGA, geoA_g5PR geoA group 5 6-FAM-ACACC GCGCTCGTTGAAGTTCCG-BHQ-1 (Lukassen et al., 2017). Total numbers of geoA were estimated as the sum the qPCR results derived from each set of primers. Total numbers of bacteria were determined by qPCR using the Mx3005P qPCR system (Stratagene) and the Brilliant III Ultra-Fast SYBR^®^ Green QPCR Master Mix (Agilent Technologies). Reactions were prepared using 30 nM ROX, 500 nM of each of the primerpair 341F/518R (CCTACGGGAGGCAGCAG and ATTACCGCGG CTGCTGG), and 1 mg⋅mL^–1^ BSA (Sigma-Aldrich) and 5 μL template DNA in 20 μL reactions (Muyzer et al., 1993).

The efficiency for both qPCR assays were always > 90% and reproducibility of triplicate pseudoreplicates always < 30%. A clear logarithmic correlation was found between the standard concentration and the *Cq* value (*R*^2^ = 0.99).

### Geosmin Analysis

Concentrations of geosmin in water from the cages were determined by standard stir bar sorptive extraction (SBSE). A commercial stir bar (Twister TM) coated with 63 μL of PDMS (length 1.00 cm and thickness of 1.00 mm) was added to 10 mL of water in a 10 mL vial, and SBSE was carried out at an ambient temperature at 1,000 rpm for 120 min. After extraction, the twisters were removed with forceps, rinsed with water, dried with lint-free tissue, and transferred to thermal desorption tubes. A calibration curve was prepared from 0, 1, 10, 50, and 100 ng⋅L^–1^ geosmin in 5% (w/vol) NaCl water and used for quantification (*R*^2^ = 0.99).

For determination of geosmin in fish flesh, a dynamic headspace analysis was employed (Petersen et al., 2014). Briefly, 10 g of fish flesh in 30 mL of water was homogenized in a 250 mL gas washing bottle, using an Ultra Turrax homogenizer (Ika). Volatile compounds from the homogenate were collected on Tenax TA traps by purging with N_2_ for 60 min at flow rate of 150 mL⋅min^–1^ at 50°C. For quantification of geosmin, fish meat samples were spiked with geosmin at 100, 250, 500, and 1000 ng⋅kg^–1^ prior to homogenization. Following the extraction, both twisters and Tenax traps were analyzed by GC-MS at conditions as described elsewhere (Petersen et al., 2014).

### Geosmin Production by Geosmin-Producing Bacterium

For evaluation of the potential contribution of geosmin from bacteria in the intestine to the content of geosmin in the fish flesh, content of free and cell-bound geosmin was determined for the geosmin-producing *Streptomyces* strain 2R, isolated from an aquaculture farm (Klausen et al., 2005). An actively growing culture of *Streptomyces* 2R was inoculated into a YEME medium (mixture of yeast extract and malt extract - YEME) and incubated for 72 h at 30°C. After 72 h of incubation, 10 mL of the culture was centrifuged (10,000 × *g* for 10 min). The supernatant was kept for analysis of extracellular and free geosmin. To measure cell-bound geosmin, the cell pellet from the centrifugation was washed with 1 × PBS buffer, followed by resuspension in 10 mL Milli-Q water. The cell suspension was heated at 100°C for 10 min to lyse the cells. After cooling, geosmin was released from the pellet and in the supernatant was measured by Twister(R) stir-bar assay as mentioned above. The number of *Streptomyces* cells was determined by epi-fluorescence microscopy after staining of homogenized cells with SYBR Green 1 (Thermo Fisher Scientific).

## Results

### Geosmin in Water and Fish

**FIGURE 1:**
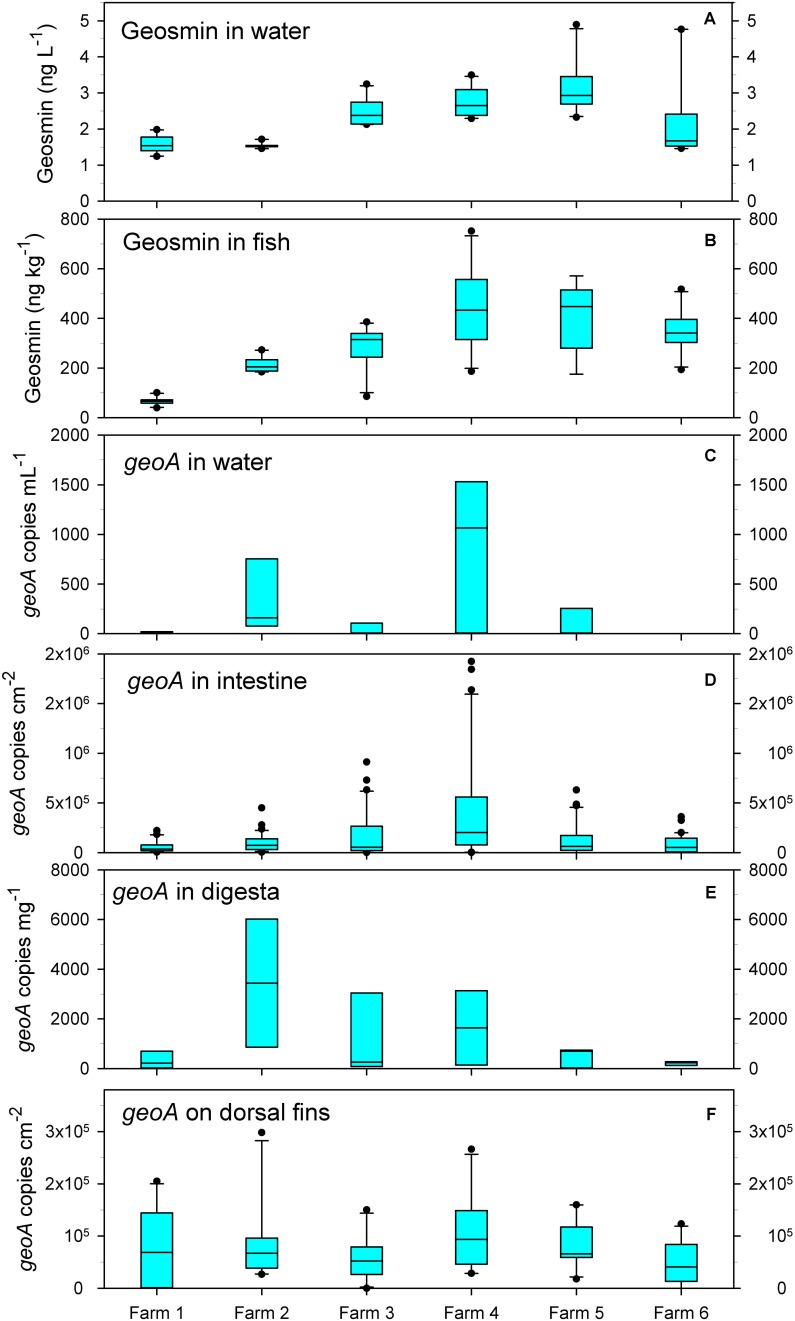
Concentrations of geosmin in water **(A)** and fish flesh **(B)**, and abundance of geoA copies in water **(C)**, intestine **(D)**, digesta **(E)**, and on the dorsal fins **(F)**. Bar show average, and boxes show 25^th^ percentile; error bars show 90^th^ and 10^th^ percentile; outliers are shown as dots.

The levels of geosmin in the water ranged from 1.2 (farm 1) to 4.9 ng⋅L^–1^ (farms 5 and 6) (mean of all farms was 2.3 ng⋅L^–1^) (Figure 1A), while it fluctuated in the fish flesh from 66 ± 15 ng⋅kg^–1^ in farm 1 to 439 ± 146 ng⋅kg^–1^ in farm 4 (mean of all fish was 284 ± 158.3 ng⋅kg^–1^) (Figure 1B). The highest concentrations of 567 and 751 ng⋅kg^–1^ were measured in fish from farm 4. The content of geosmin in water and fish co-varied and had a positive correlation (Pearson; *r* = 0.66).

### Quantification of *geo*A

The number of *geoA* copies (reflecting the potential for geosmin production) varied significantly in the water within and between the farms, but large variations were also found within the intestinal mucous layer, digesta, and on the dorsal fin of the individual fish.

In the water, the *geoA* number varied from 0 to 1531 copies⋅mL^–1^. The lowest average numbers (<250 copies⋅mL^–1^) occurred in farms 1, 3, and 5 (Figure 1C). A generally low number of bacteria was present in the intestinal mucous and on the dorsal fins, and combined with high levels of host DNA, lead us to determine the *geoA* copy numbers per total surface area sampled. In the fish intestinal mucous layer, the average number of *geoA* copies ranged from 5.8⋅10^4^ cm^–2^ (fish in farm 1) to 4.32⋅10^5^ cm^–2^ (fish in farm 4) (Figure 1D). In digesta in the intestine, the *geoA* copies were highest in fish from farm 2 (average of 3440 copies⋅g^–1^) and lowest in fish from farm 6 (average of 210 copies⋅g^–1^) (Figure 1E). On the dorsal fin, the average number of *geoA* copies was rather similar, ranging from 4.9⋅10^4^ (farm 6) to 1.05⋅10^5^ copies⋅cm^–2^ in (farm 4) although large variations occurred (Figure 1F).

Numbers of *geoA* copies in the intestinal mucous layer and geosmin in the water co-varied between the farms (*r* = 0.309; *p* = 0.02), and *geoA* numbers in the digesta had a positive correlation to geosmin in the fish (*r* = 0.169) and in the water (*r* = 0.227), however not statistically significant (both *p* > 0.1).

### *geoA* Copies Relative to the Total Bacterial Density

Assuming that all geosmin-producing bacteria only carry one copy of the *geoA* gene, or a similar number of copies, the ratio of geosmin-producing bacteria relative to the total number of bacteria were similar in the water, on dorsal fins, and in intestinal mucous and digesta. The proportion of geosmin producers constituted up to 2.1% of the bacterial populations (Table 1). The highest percentage of geosmin-producing bacteria was found in the digesta (up to 2.12%), as compared to intestinal mucous (up to 0.44%) and the dorsal fins (up to 0.40%). In the water, up to 0.64% of the bacteria were determined to carry the *geoA* gene.

**TABLE 1:** Proportion of *geoA* gene copies relative to the total bacterial density (number of OTUs).

**Fish farm**	**Water**	**Intestinal mucous surface**	**Digesta**	**Dorsal fin surface**
1	0.0–0.64%	0.04–0.22%	0.002–2.12%	0.0–0.40%
2	0.012–0.15%	0.04–0.98%	0.0–0.14%	0.015–0.14%
3	0.0–0.038%	0.0–0.22%	0.001–0.45%	0.0–0.22%
4	0.14–0.45%	0.0–0.29%	0.0–0.02%	0.0–0.15%
5	0.0–0.009%	0.0–0.33%	0.0–1.78%	0.02–0.14%
6	0.0%	0.0–0.44%	0.001–0.004%	0.0–0.08%

*Range for each of the six fish farms shown. Average 16S rRNA gene copies were 6.3⋅10^5^ mL^–1^ water, 1.1⋅10^9^ cm^–2^ intestinal mucous, 1.2⋅10^7^ mg^–1^ intestinal digesta, and 1.1⋅10^5^ cm^–2^ skin of the dorsal fins.*

**FIGURE 2:**
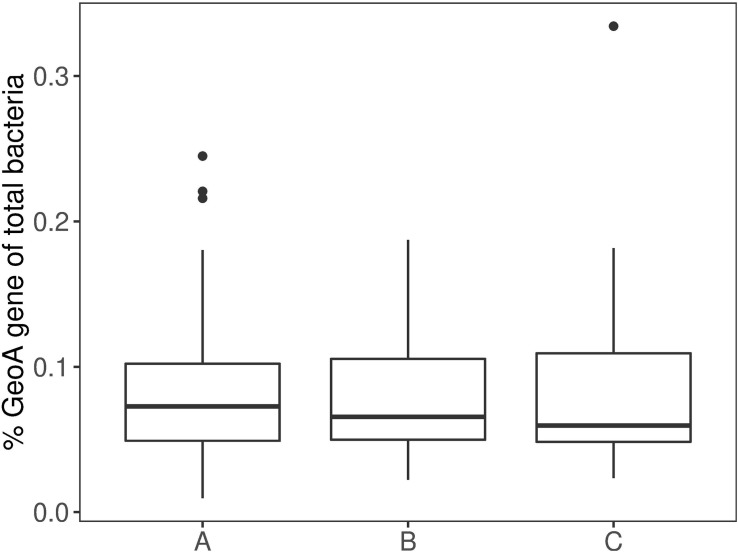
Boxplot of differences between the intestinal mucous (A) (*n* = 54), (B) (*n* = 52) and (C) (*n* = 54). Boxes show 25^th^ percentile; error bars show 90^th^ and 10^th^ percentile; outliers are shown as dots.

Analysis of the longitudinal distribution of geosmin producers at three sections of the intestine did not show variations in the number of *geoA* copies, relative to the total bacterial density (one-way ANOVA with Tukey’s HSD *post hoc* test) (Figure 2). Apparently, geosmin-producing bacteria made up a rather similar proportion of the intestinal microflora of about 0.1%.

### Microbial Communities

The amplicon sequencing of the V1–V3 region of the 16S rRNA gene yielded a total of 5,558,169 sequences with an average number of 28,799 ± 15,415 per sample (mean ± SD). The microbial diversity, determined from the number of observed OTUs and the diversity index Chao1, was found to vary between sample type (water, skin and intestinal mucous, and digesta), but was rather similar between the fish farms for each sample type. An average number of 715 ± 217, 153 ± 122, 65 ± 79, and 51 ± 46 OTUs was observed across the fish farms for water, skin, intestinal digesta, and mucous samples, respectively. Estimated species richness (Chao1) was determined to an average of 581 ± 489 OTUs for all samples.

**FIGURE 3:**
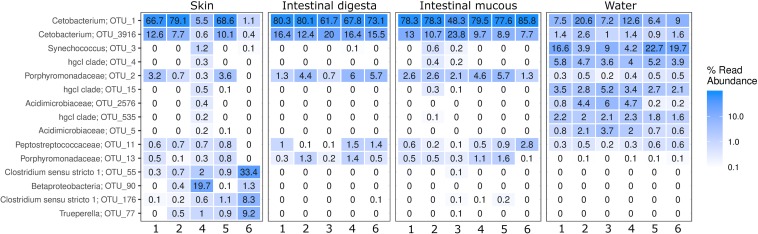
Heatmap showing the microbial community composition in samples from skin, intestinal digesta and intestinal mucous and surrounding water. The 15 most abundant groups shown as the proportion of the reads with their taxonomic classification listed at the highest resolution possible.

A heatmap of the 15 most abundant OTUs in all farms and sample types shows that the microbial populations were dominated by *Fusobacteria*, *Cyanobacteria*, *Bacteroidetes*, *Firmicutes*, *Actinobacteria*, and *Proteobacteria* (Figure 3). *Cetobacterium* spp. was the most abundant and common genus in the four sample types (1.1–85.8% of total reads), but the cyanobacterium *Synechococcus* also had a high abundance in the water (3.9 – 22.7% of total reads). In addition, *Actinobacteria* (represented by three clades of *Actinomycetales*) appeared more frequent and abundant in the water than in the other environments. On the dorsal fins, *Cetobacterium*, *Clostridium*, *Betaproteobacteria*, and *Trueperella* made up the majority of the abundant groups. In content and mucous of the intestine, the family *Porphyromonadaceae* was abundant, but less frequent than *Cetobacterium*. Samples from the intestinal mucous layer and dorsal fin were excluded, due to lack of sufficient sequences from farms 3 and 5, respectively.

Among the detected taxonomic groups, the following putative geosmin-producing bacteria were found: *Actinomycetales*, *Cyanobacteria*, *Myxococcales*, and *Sorangium* (Table 2). In the water, *Actinomycetales* was an abundant group and made up 14.8–24.3% of the total OTUs. The proportion of this order was lower in digesta, intestinal mucous, and dorsal fin, and it only constituted 0.01–9.3% of the identified taxa. *Cyanobacteria* occurred in both digesta, intestinal mucous, and on the dorsal fins at abundances of 0.01–3.5% but were more abundant in the water (range of 9.2–27.7% of the OTUs). *Myxococcales* made up 0.2–3.7% on the dorsal fins, but only 0.01–0.9% in water, digesta, and intestinal mucous. *Sorangium* constituted less than 0.01% of the reads in all samples, except for the dorsal fins, where this genus ranged from 0.01 to 0.1%. No *Streptomyces* were detected in any of the analyzed samples.

**TABLE 2:** Potential *geoA* containing groups as detected by 16S rRNA gene amplicon sequencing.

**Sample type**	***Actinomycetales***	***Streptomyces***	***Cyanobacteria***	***Myxococcales***	***Sorangium***
Water	14.8–24.3%	0.0%	9.2–27.7%	0.3–0.8%	> 0.01%
Intestinal digesta	> 0.01%	0.0%	> 0.01–0.5%	> 0.01–0.1%	> 0.01%
Intestinal mucous	> 0.01–0.2%	0.0%	> 0.01–1.8%	> 0.01–0.9%	> 0.01%
Dorsal fin	>0.01–9.3%	0.0%	0.1–3.5%	0.2–3.7%	> 0.01–0.1%

*Relative abundance and range are shown.*

### Geosmin-Producing Bacteria

**FIGURE 4:**
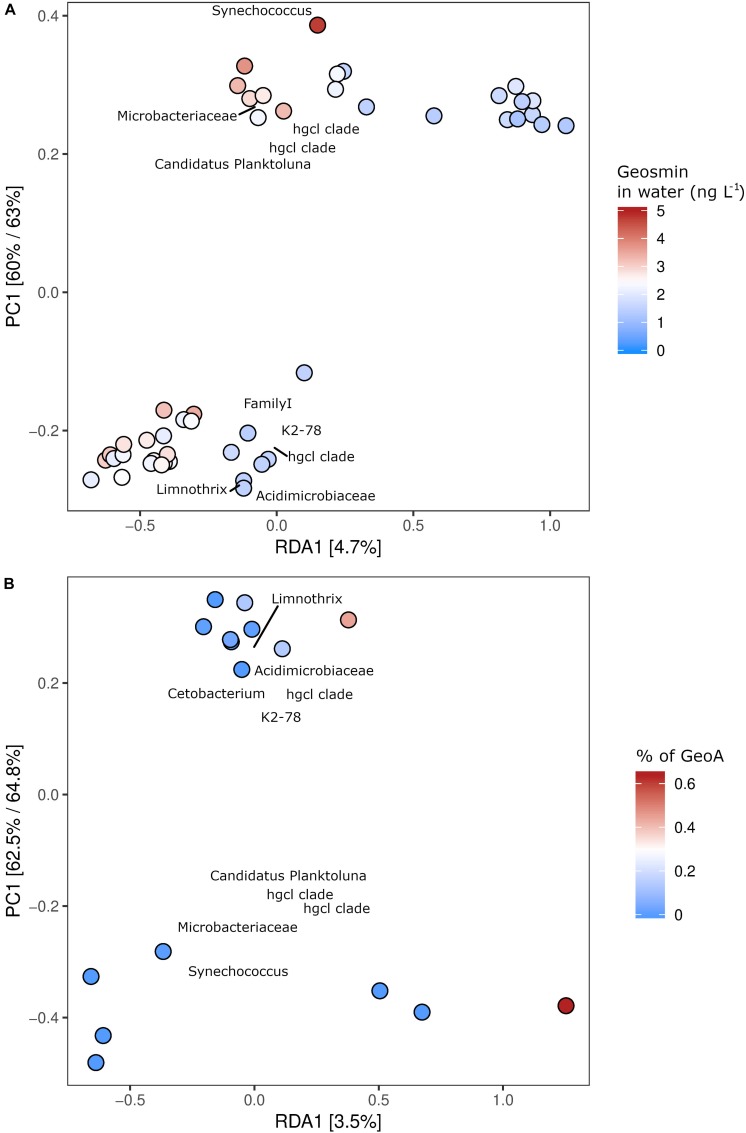
Relationship between content of geosmin in the water phase and OTU abundance in the six fish farms, as explored by a constrained RDA analysis **(A)**. The relationship between the content of geoA copies in the water phase and OTU proportion in the fish farms by a constrained RDA analysis **(B)**. Both **(A,B)** show two geographical grouping of the OTU abundances (top and lower part of panels of the examined fish farms) and a gradient related to the geosmin levels along the RDA1 axis. The 10 OTUs with the strongest correlation (loading) are shown on the plot, with their taxonomic classification listed at the highest resolution possible.

The microbial taxa with the strongest correlation (loading) to the content of geosmin in the water and the fish biosphere from all six fish farms were identified through redundancy analysis (RDA) (Figures 4A,B). The 10 OTUs with the strongest loading within the model to geosmin levels or the *geoA* copy numbers were identified. The analysis revealed that between 3.5 and 4.7% of the microbial community variation could be explained by the constrained parameters. In both models, the samples clustered together geographically in two groups consisting of farms 2, 3, and 4 (top of Figure 4A) and farms 1, 5 and 6 (lower part of Figure 4A), with secondary clustering based on the tested parameter. It also showed that the *Actinomycetales* group hgcl-clade, *Acidimicrobiaceae, Candidatus* Planktoluna and *Microbacteriaceae* (all *Actinobacteria*) together with the cyanobacterial *Synechococcus, Limnotrix*, and an unidentified *Cyanobacterium* (FamilyI) were microbial taxa with the strongest loading to the geosmin levels. For mucous samples, the ordination analysis identified the *Actinomycetales* hgcl clade, *Synechococcus*, and *Deltaproteobacteria* with the highest co-occurrence to geosmin (data not shown). *Trueperella* and *Microthrix* (both *Actinomycetales*) were identified as strong loading organisms for the geosmin levels in the tissue for intestinal digesta and skin samples. Furthermore, *Trueperella* was found to strongly correlate with the *geoA* levels on the skin (data not shown).

To evaluate a potential bacterial release of geosmin in the intestine, the geosmin-producing strain *Streptomyces* 2R (isolated from a fish farm) was used as a proxy. The measured extracellular and cell-bound amounts of geosmin were 73⋅10^–18^ and 67⋅10^–18^ g⋅cell^–1^, respectively. Assuming that all the calculated geosmin-producing cells (in water and associated with the fish) produced free and cell-bound geosmin similar to *Streptomyces* 2R, the potential contributions of geosmin to water and fish were estimated. In the water, the geosmin-producing bacteria may have released from 0.01 to 1.21 ng⋅L^–1^ (Table 3). Relative to presence of geosmin in the water, this contribution varies from insignificant (farm 1) to 44% of geosmin in the water (farm 4). In the intestinal mucous, the geosmin producing bacteria may have released from 66 to 438 ng geosmin per fish intestine (Table 3). If all of this geosmin were absorbed by the fish, it would represent between 26.8 and 47.6% of the geosmin content in the flesh. Contributions of geosmin from the digesta were estimated to account for similar levels to the geosmin content in the fish flesh, but with significantly higher deviations due to the varying content of digesta. The importance of the bacteria on the skin surface to the geosmin content in the fish flesh remains to be explored due to uncertainties related to water currents and assumptions concerning the uptake efficiencies.

**TABLE 3:** Potential contribution of geosmin by geosmin-producing bacteria in water, intestinal mucous, digesta and on dorsal fin.

	**Intestinal mucous**	**Flesh**		**Digesta**	**Fins**	**Water**	
**Farm**	**Calculated geosmin production**	**Measured geosmin in flesh**	**Potential geosmin contribution by bacteria in intestinal mucous to content in flesh**	**Calculated geosmin production**	**Calculated geosmin production**	**Calculated geosmin production**	**Measured geosmin**
	**ng ⋅ intestine^–1^ ± SE**	**ng ⋅ kg^–1^ ± SE**	**% (average)**	**pg ⋅ g^–1^ ± SE**	**ng ⋅ cm^–2^ ± SE**	**ng ⋅ L^–1^ ± SD**	**ng ⋅ L^–1^**
1	325.9	664.9	47.7	5.94.0	1.450.47	0.010.01	1.50.3
2	6410.9	21510.2	29.5	67.450.6	1.720.50	0.460.30	1.50.1
3	13633.0	28926.5	46.9	22.118.8	1.140.26	0.050.05	2.50.4
4	18642.9	43951.9	42.3	32.129.3	2.060.46	1.210.61	2.70.4
5	11426.1	40865.4	27.9	9.44.7	1.650.28	0.120.12	3.20.7
6	9419.3	35127.1	26.8	4.10.9	0.960.25	0	0

*Assumptions were: (1) Each geosmin producing bacteria released 140 × 10^–18^ g geosmin as observed for *Streptomyces* 2R; (2) the OTUs has been affiliated into the groups determined in Table 2; (3) for calculation of geosmin release from the entire intestine, surface area of the intestine in tilapia was assumed to be 2000 cm^2^ (estimated from Frierson and Foltz, 1992). Weight of fish from each of the six farms (range 250 to 651 g) was used to convert potential fish-specific geosmin contribution by intestinal bacteria to weight-specific geosmin content in the flesh. Measured geosmin content in water and fish flesh refers to values shown in Figure 1. SE, standard error; SD, standard deviation.*

## Discussion

The present findings of geosmin and geosmin-producing bacteria in water and fish from net cages in the Brazilian fresh water reservoirs show that these floating production systems are also exposed to occurrence of off-flavors, as observed for fish production in freshwater tanks, e.g., production of tilapia and arctic char (Guttman and van Rijn, 2008; Houle et al., 2011). Identification of the major sources and habitats of the off-flavor producing microorganisms is required to better understand, and potentially reduce, the mechanisms governing fish tainting in aquatic ecosystems.

### Geosmin in Water and Fish

Concentrations of geosmin in the water (1.2–4.9 ng⋅L^–1^) were comparable to concentrations measured in combined pangasius-tilapia ponds in Bangladesh (Petersen et al., 2014), but were significantly lower than concentrations in tilapia ponds in Thailand (0.41–2.33 μg⋅L^–1^; Gutierrez et al., 2013). Despite the relatively low levels of geosmin found in the Brazilian farms, the content of geosmin in the fish flesh (mean of 284 ng⋅kg^–1^, but up to 751 ng⋅kg^–1^ was measured) was probably above the human threshold for detection of geosmin, at least when compared to the threshold observed for rainbow trout flesh (250 ng⋅kg^–1^) (Petersen et al., 2011). The threshold for geosmin detection in tilapia remains to be determined, but it may differ from rainbow trout due to different fat content and presence of other flavors in tilapia.

The observed positive correlation between geosmin in water and fish might indicate that geosmin dissolved in the water was the primary geosmin source in the flesh due to uptake via the gill surface (From and Hørlyck, 1984). However, other and potential sources and dissemination routes of geosmin may also be important. Here, we detected geosmin-producing bacteria (quantification of *geoA* by qPCR), not only in the water, but also in the intestinal mucous, digesta, and on skin of the dorsal fin.

### Microbial Community Structure and Geosmin Producers in the Water

The microbial community identified in the water from the net cages revealed rather complex communities with 715 different OTUs. The most abundant phyla were *Fusobacteria*, *Cyanobacteria*, *Bacteroidetes*, *Firmicutes*, *Actinobacteria*, and *Proteobacteria*, which resemble microbial communities reported in other epilimnetic waters of lakes worldwide (Newton et al., 2011). Most of the identified microorganisms have not been characterized with respect to specific functions in freshwater, and only few have been directly associated with specific environments, e.g., *Cetobacterium* (*Fusobacteria*) and *Porphyromonadaceae* (*Bacteroidetes*) have been associated to fecal matter from fish (Austin, 2006; Tsuchiya et al., 2008). Possibly, presence of these bacteria in the Brazilian waters may indicate fecal contamination by the high densities of tilapia in the cages.

*Synechococcus* and other *Cyanobacteria* were also abundant in the water and may reflect periods of high nutrient levels in the water (Downing et al., 2001). *Synechococcus* is commonly found in open freshwater reservoirs and has been associated with geosmin production (Billica et al., 2010), and may have been a potential source of geosmin in the present fish farms. Several other putative geosmin-producing bacteria were identified in the water, e.g., groups affiliating within *Actinomycetales*, such as the genus *Trueperella* and the family *Acidimicrobiaceae* (Llirós et al., 2014; Lukassen et al., 2017). Geosmin synthases are found in almost all *Actinomycetales* (Yamada et al., 2015). *Trueperella* was most abundant on the dorsal fin, while a representative of *Acidimicrobiaceae* was dominant in the water. Within the class of *Deltaproteobacteria*, members of *Myxococcales* are also known to harbor several geosmin producers (Dickschat et al., 2005). A few other *Deltaproteobacteria* were observed, but cannot be classified higher than to the class level and might therefore also be potential geosmin producers. No *Streptomyces* were detected in any of the environments, suggesting that these geosmin producers (Klausen et al., 2005) were not common bacteria in the reservoirs.

Taxonomic groups determined from amplicon sequencing of the 16S rRNA gene that harbor geosmin producers may also include species that do not produce geosmin, and thus, prediction of geosmin production from analysis of taxonomic groups might be severely biased. Therefore, a constrained ordination approach was carried out to link the presence of putative geosmin producers to the sites with the highest concentrations of geosmin and *geoA* gene copies. The constrained ordination by redundancy analysis (RDA) was conducted on the 16S rRNA gene sequencing data, using geosmin content and the total *geoA* copy numbers in the water as the constrained factors. Despite relatively small numbers of *geoA* gene copies and low geosmin levels in the fish farms, the RDA approach identified putative geosmin-producing bacteria primarily as the most important organisms responsible for occurrence of the geosmin in the farms (Figure 4A). In water from the net cages, OTUs affiliating with *Actinobacteria* and *Cyanobacteria* correlated positively with both the geosmin levels and the numbers of *geoA* copies (Figure 4B), suggesting that these taxa were major contributors of geosmin. The ubiquitously present *Cetobacterium* was not considered as a putatively geosmin-producing bacterium, since its correlation is most likely based on its high read abundance.

The identified OTUs with the highest loading for the presence of geosmin belonged to the *Actinomycetales* group, e.g., the hgcl clade, but also other taxonomic groups previously described to contain geosmin-producing bacteria such as *Acidimicrobiaceae*, *Candidatus* Planktoluna, cyanobacterial representative *Familyl*, and *Synechococcus*. *Candidatus* Planktoluna and the unclassified *Cyanobacteria* were not included in the 15 most abundant OTUs (Figure 3). *Actinomycetales*, *Microbacteriaceae*, and cyanobacterium *Limnotrix* also correlated with the levels of *geoA* gene copies in the water (Figure 4B). All of these clades affiliate within the order of *Actinomycetales* or the *Cyanobacteria* phylum and are therefore assumed to be the dominant geosmin producers in the water (Ma et al., 2013; Lukassen et al., 2017).

### Geosmin-Producing Bacteria in Intestine, Digesta, and on Dorsal Fins

The taxonomic affiliations of the potential geosmin-producing bacteria in the intestinal mucous layer and digesta were similar to those from the water and also included geosmin producers affiliated to *Cyanobacteria*. The co-occurrence between geosmin producers in water and fish biosphere might reflect that these bacteria were ingested with fish feed and subsequently occurred in the gastrointestinal tract. Yet, actively feeding on phytoplankton is also possible due to an efficient mucous trap mechanism and pharyngeal teeth for filter feeding in tilapia (Moriarty and Moriarty, 1973; Moriarty et al., 1973). In general, tilapia appears to be an opportunistic and possibly omnivorous fish that has been shown to feed on both detritus and phytoplankton (Gutierrez et al., 2013). A special characteristic for Nile tilapia is its capability to differentiate between toxic and non-toxic *Cyanobacteria* and interrupt the filter feeding when toxic species are present.

During digestion of *Cyanobacteria* and other phytoplankton, enzymes in the intestinal system (trypsin, chymotrypsin, and pancreatic α-amylase) and the low stomach pH may have facilitated lysis and release of intracellular geosmin (Moriarty, 1973). Calculation of the potential release of geosmin by geosmin-producing bacteria in the intestinal mucous showed that a substantial portion (27 to 48%) of the geosmin content in the fish flesh might have originated from bacteria in the intestine (Table 3). However, more knowledge on the activity of these potential geosmin producers is required to conclude on release of geosmin by intestinal bacteria and uptake in the flesh. The degree of the geosmin-producing bacteria lysed during passage in the intestinal system remains unknown. Also, actual content of geosmin production by the most abundant geosmin producers in the intestine needs to be determined. Nevertheless, this study has shown for the first time that intestinal bacteria have potential to taint fish in aquaculture farms.

Digesta in the intestine may also have contributed to the presence of geosmin in the flesh, but the calculated free and cell-bound geosmin was about 1,000-fold lower per g than in the mucous layer. Geosmin-producing bacteria on the skin, as exemplified by the dorsal fin, may have released geosmin to the water, but was probably unimportant to the content in the flesh due to the relatively small numbers, compared to the digesta. Uptake of geosmin has been estimated to be 40 times slower across the epithelial lining than transport across the gills (From and Hørlyck, 1984).

The high proportion of putative geosmin producers affiliating with *Actinomycetales* and *Cyanobacteria* identified in the intestinal mucous layer, combined with the positive correlations to geosmin and *geoA* copies in water and mucous layer all support the hypothesis that the digestive system might be an important source of tainting of the fish. The presence of *Actinomycetales* and other putative geosmin producers in the intestine was unexpected. Possibly, some of these bacteria grow in the intestinal mucous layer, but absorption from active or lysed geosmin producing bacteria in the digesta cannot be excluded.

The observation of geosmin-producing bacteria in the intestine has important implications for strategies to reduce off-flavor problems in fish breeding. As an alternative to the present depuration of fish in clean water for several days (Howgate, 2004), manipulation of the gut microflora by application of probiotic bacteria might be a future strategy for improving the taste of freshwater fish from aquaculture facilities.

In summary, the present study detected and identified potentially geosmin-producing organisms in Brazilian aquaculture by combining 16S rRNA gene sequencing, qPCR, and measurements of the geosmin levels. The proportion and composition of potentially geosmin-producing bacteria among all bacteria appear similar in the water phase, digesta, mucous layer of the gastrointestinal tract, and on the skin. However, differences in bacterial densities suggest that uptake through the intestinal tract may be a potential major, but hitherto overlooked, route of geosmin uptake by the fish in aquaculture systems.

## Data Availability Statement

The datasets generated for this study can be found in the European Nucleotide Archive (Accession Number: PRJEB33049).

## Ethics Statement

Ethical review and approval was not required for the animal study because animals were sampled as part of the daily aquaculture procedure. Written informed consent was obtained from the owners for the participation of their animals in this study.

## Author Contributions

ML and JN designed the study. SB performed the fieldwork. RP and MP measured the geosmin. ML and NJ performed all the bioinformatic analyses. ML and JN wrote the manuscript with contributions from NOJ, GD, and RS.

## Conflict of Interest

The authors declare that the research was conducted in the absence of any commercial or financial relationships that could be construed as a potential conflict of interest.

## Footnotes

1 www.rstudio.com
